# Trafficking, localization and degradation of the Na^+^,HCO_3_^−^ co-transporter NBCn1 in kidney and breast epithelial cells

**DOI:** 10.1038/s41598-018-25059-7

**Published:** 2018-05-09

**Authors:** Christina Wilkens Olesen, Jens Vogensen, Ida Axholm, Marc Severin, Julie Schnipper, Isabella Skandorff Pedersen, Jakob Hjorth von Stemann, Jacob Morville Schrøder, Dan Ploug Christensen, Stine Falsig Pedersen

**Affiliations:** Section for Cell Biology and Physiology, Department of Biology, University of Copenhagen, Universitetsparken 13, DK-2100 Copenhagen Ø, Denmark

## Abstract

The Na+;HCO3^−^ co-transporter NBCn1 (SLC4A7) is a major regulator of intracellular pH yet its trafficking and turnover are essentially unstudied. Here, we used MDCK-II and MCF-7 cells to investigate these processes in epithelial cells. GFP-NBCn1 membrane localization was abolished by truncation of the full NBCn1 C-terminal tail (C-tail) yet did not require the C-terminal PDZ-binding motif (ETSL). Glutathione-S-Transferase-pulldown of the C-tail followed by mass spectrometry analysis revealed putative interactions with multiple sorting-, degradation- and retention factors, including the scaffolding protein RACK1. Pulldown of FLAG-tagged deletion constructs mapped the RACK1 interaction to the proximal NBCn1 C-tail. Proximity Ligation Assay and co-immunoprecipitation confirmed that native NBCn1 interacts with RACK1 in a cellular context. Consistent with a functional role of this complex, RACK1 knockdown reduced NBCn1 membrane localization without affecting total NBCn1 expression. Notably, only non-confluent cells exhibited detectable NBCn1-RACK1 plasma membrane co-localization, suggesting that RACK1 regulates the trafficking of NBCn1 to the membrane. Whereas total NBCn1 degradation was slow, with a half-life of more than 24 h, one-third of surface NBCn1 was constitutively endocytosed from the basolateral membrane within 60 min. This suggests that a fraction of NBCn1 exhibits recycling between the basolateral membrane and intracellular compartment(s). Our findings have important implications for understanding NBCn1 regulation as well as its dysregulation in disease.

## Introduction

The electroneutral Na^+^;HCO_3_^−^ co-transporter NBCn1 (SLC4A7) is a member of the SLC4 family of bicarbonate transport proteins and is a major mediator of net cellular acid extrusion in most tissues studied^1,2^. NBCn1 is widely expressed in many human organs and plays essential roles for their normal physiological function. In turn, NBCn1 dysfunction has been linked to cardiovascular diseases and more recently to breast cancer^1,3–5^. Thus, NBCn1 expression is increased in at least some human breast cancer tissues compared to normal tissue^6,7^, NBCn1 knockout mice exhibit reduced breast tumor development after chemical carcinogenesis^8^, and stable knockdown of NBCn1 reduces xenograft growth of human breast cancer cells in immunosuppressed mice^7^. We have demonstrated that NBCn1 transcription in human breast cancer cells is controlled by oncogenic human epidermal growth factor receptor 2 (p95HER2) signaling via the transcription factor Krüppel like factor 4 (KLF4), downstream from phosphatidylinositol-3 kinase (PI3K)/Akt and Ras/Raf/MEK/ERK activation^9^. Furthermore, expression of the p95HER2 receptor also increased NBCn1 mRNA stability^10^.

Bioinformatic analysis and comparison with the recent crystal structure of the Cl^−^/HCO_3_^−^ exchanger 1 (AE1)^11^ suggests a membrane topology for NBCn1 with 14 transmembrane domains, a long, structured N-terminal and a short C-terminal intracellular domain terminating in a PDZ-binding motif (-ETSL)^2,12^. The NBCn1 protein likely forms homodimers in the membrane^2^. The C-terminal PDZ-binding motif was found to link NBCn1 to the Na^+^/H^+^ exchange regulatory factor 1 (NHERF-1, EBP50)^13^, the postsynaptic density protein 95 (PSD-95)^14^, and, indirectly, to the V-type H^+^-ATPase^15^ and the cystic fibrosis transmembrane regulator (CFTR)^16^.

Sorting of membrane proteins is a multistep process involving (i) initial sorting in the endoplasmic reticulum (ER), passage through the *trans*-Golgi Network (TGN), and insertion into correct positions in the membrane; (ii) membrane retention; and (iii) recycling or degradation of the membrane protein via either clathrin-dependent or –independent endocytosis^17^. Tightly controlled membrane protein sorting is pivotal for the function of epithelial cells. Disturbances in the cellular polarity program are integral to the development of epithelial cancers^18,19^. Additionally, mistargeting or defective membrane localization of specific membrane proteins plays important roles in many diseases including cystic fibrosis^20^ and kidney diseases^21^.

Essentially nothing is known about the mechanisms of membrane targeting and sorting of NBCn1. In the duodenal epithelium, NBCn1 is predominantly basolateral^22^. In some parts of the choroid plexus, NBCn1 is found at the apical membrane^23^, and truncation of the entire C-terminal was found to abolish NBCn1 membrane targeting in HEK293 cells^24^. Thus, it appears that NBCn1 sorting mechanisms differ between different epithelial cells, and that at least part of the signal(s) for membrane targeting reside in the C-terminal. PDZ-binding motifs play important roles in sorting and retention of membrane proteins in epithelial cells^25^, yet truncation of the PDZ-binding motif did not attenuate plasma membrane NBCn1 activity in HEK293 cells^26^.

The kinetics and mechanisms of membrane retention, recycling and degradation of NBCn1 are poorly understood. NBCn1 membrane expression in parotid acinar (ParC5) cells was reported to be very stable compared to that of NBCe1 (SLC4A4, an electrogenic SLC4 family member), suggesting that it is not constitutively endocytosed^27^. However, we recently noted that upon treatment of MCF-7 breast cancer cells with the chemotherapeutic drug cisplatin, NBCn1 lost its membrane localization and appeared in a punctate perinuclear localization, whereas the membrane localization of the Na^+^/H^+^ ion transporter NHE1 was unaltered^28^. This suggests the existence of distinct endocytic mechanisms for NBCn1, which can be activated under some conditions.

In this study, we identify novel aspects of the trafficking and localization of NBCn1 in epithelial cells. We carried out Glutathione-S-Transferase (GST)-pulldown of the full length NBCn1 C-terminal tail, followed by mass spectrometry analysis (MS) to identify putative C-terminal binding partners. This revealed multiple interacting proteins of relevance to trafficking and membrane targeting/retention, including the small scaffolding protein Receptor for Activated C Kinase-1 (RACK1), which we demonstrate interacts with NBCn1 and regulates its membrane localization. Using Madin-Darby Canine Kidney II (MDCK-II) epithelial cells and MCF-7 human breast cancer epithelial-derived cells, we find that NBCn1 protein turnover was very slow, with a half-life of 24–76 h. Finally, we show for the first time that NBCn1 is rapidly constitutively endocytosed from the basolateral membrane in these cells. This work is the first detailed analysis of the kinetics and mechanisms of NBCn1 targeting, sorting, and retrieval in epithelial cells and contributes to a broader understanding of how NBCn1 is regulated in these cells, which will also advance understanding of its dysregulation in diseases.

## Results

### Native NBCn1 localizes to the basolateral membrane in polarized MDCK-II and MCF-7 cells

**Figure 1:**
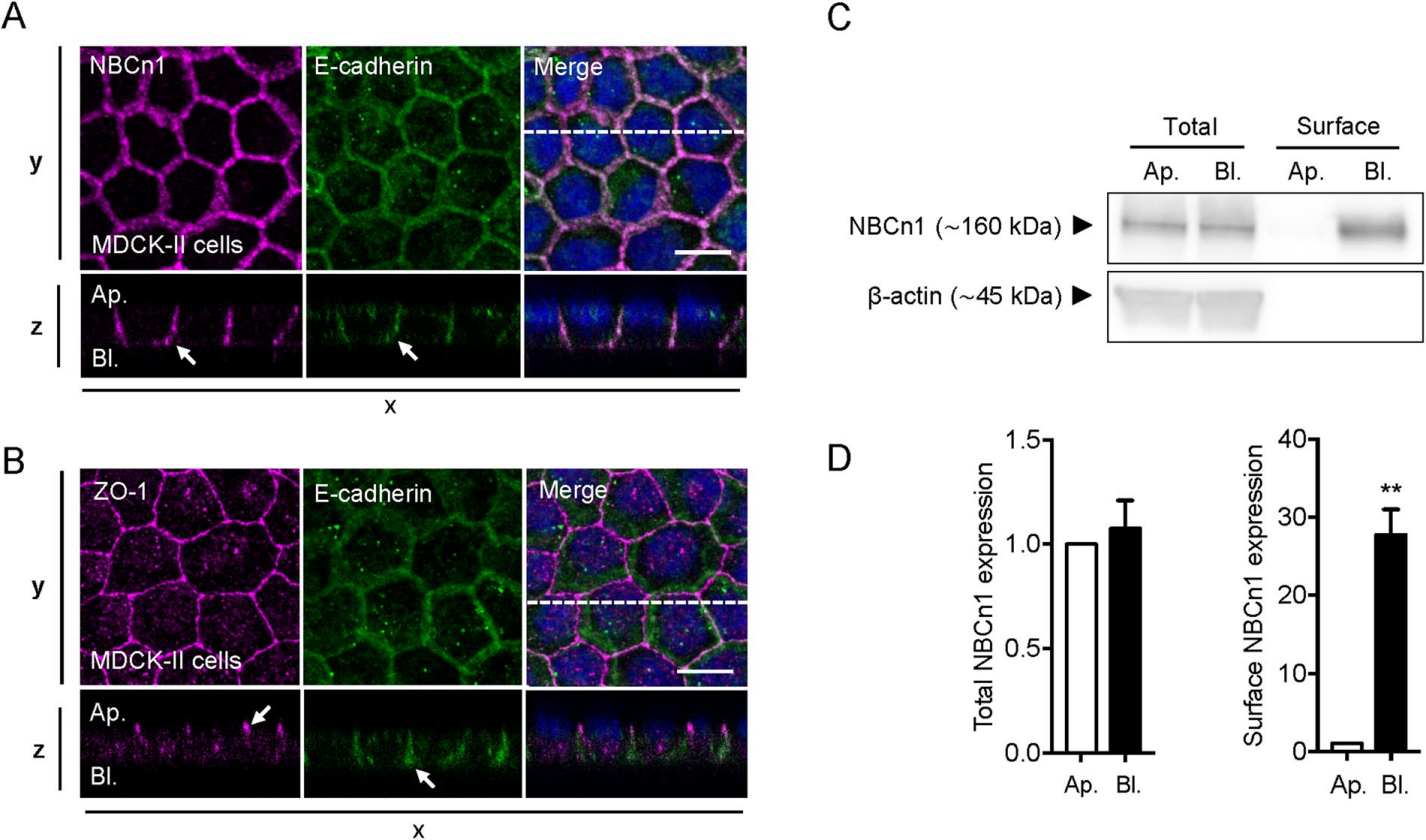
NBCn1 localizes to the basolateral membrane in polarized MDCK-II cells. MDCK-II cells were cultured on Transwell filters for 4 days to allow polarization (**A**–**D**). Cells were lysed and processed for immunofluorescence analysis (**A**,**B**) or cell surface biotinylation followed by Western blotting (**C**,**D**). (**A**,**B**) fluorescence images of NBCn1 (magenta), E-cadherin (green) and ZO-1 (magenta). Nuclei stained with DAPI (blue). Images were collected as z-stacks on a confocal microscope and shown as z-projections with corresponding xz-scans. Scale bar 10 µm. (**C**) Representative Western blots. ß-actin was used as a loading control. (**D**) Quantification of total NBCn1 expression and NBCn1 surface expression. Values are normalized to the apical pool of NBCn1. Quantifications of Western blot data are shown as means with SEM error bars. ** indicate P < 0.01. Student’s *t* test. Data are representative of 3 independent experiments. Ap.: apical; Bl.: basolateral.

NBCn1 has previously been shown to localize *in situ* to the basolateral surface of human duodenal villus cells^22^. To determine the NBCn1 localization in epithelial MDCK-II cells, cells were cultured on Transwell filters for 4 days to allow polarization. Cells were fixed and subjected to immunofluorescence analysis of subcellular localization by confocal imaging (Fig. 1A,B). Zona occludens protein 1 (ZO-1) and E-cadherin were used as markers of tight junctions (apical) and of the basolateral domain, respectively^29^. ZO-1 and E-cadherin showed clear localization to the tight junction- and basolateral regions, respectively (Fig. 1B; arrowheads), suggesting proper polarization of the MDCK-II cells under these conditions. NBCn1 strongly co-localized with E-cadherin, consistent with its expected basolateral localization (Fig. 1A). Further, the X-Z-scan seen in Fig. 1A suggests a more lateral than basal localization of NBCn1. A similar pattern of NBCn1 basolateral localization was found in polarized epithelial MCF-7 breast cancer cells cultured on Transwell filters (Fig. S1). To substantiate that NBCn1 is indeed basolaterally localized, we performed separate apical and basolateral biotinylation of Transwell-polarized MDCK-II cells, followed by lysis, streptavidin-pull-down, and Western blotting (Fig. 1C,D). NBCn1 was exclusively detected in the basolateral pull-down fraction (p < 0.01; Fig. 1C,D).

These results show that NBCn1 localizes to the basolateral membrane of MDCK-II and MCF-7 epithelial cells.

### Deletion of the NBCn1 C-terminal, but not of the PDZ-binding motif alone, abolishes NBCn1 plasma membrane localization

**Figure 2:**
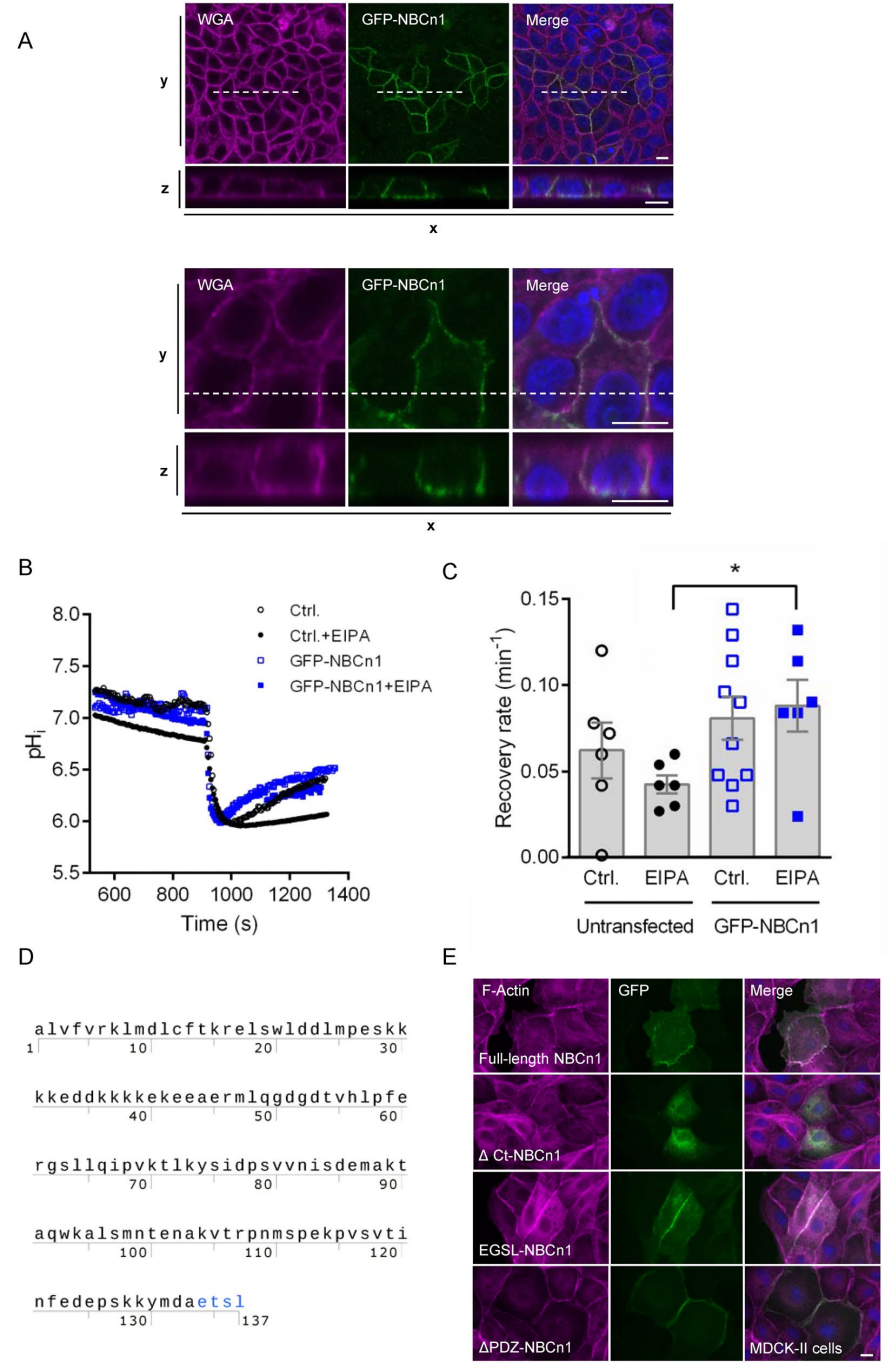
GFP-NBCn1 membrane localization and function. (**A**) GFP-NBCn1 localizes to the basolateral cell surface. MDCK-II cells transiently transfected with GFP-NBCn1 were cultured to 100% confluency and maintained in culture for 12 days before fixation. Cells were stained with wheat germ agglutinin (WGA) and DAPI prior to confocal imaging. Bottom rows are xz-scans of the dashed line shown in top rows (xy-scans). Upper and lower panel are low- and high magnification images, respectively. Scale bars: 10 µm. Representative images from 4 independent experiments are shown. (**B**,**C**) GFP-NBCn1 is a functional net acid extruder. Capacity for net acid extrusion after an NH_4_Cl-prepulse-induced intracellular acidification was assessed by live imaging of BCECF fluorescence in GFP-NBCn1-transfected and control MCF-7 cells, in absence and presence of the NHE1 inhibitor EIPA. B, representative traces, C, Mean recovery rates. *p < 0.01, Student’s *t*-test, n = 6–10 per condition. (**D**) Amino acid sequence of the human NBCn1 C-terminal tail (Met^1111^-Leu^1214^), showing the PDZ-binding ETSL motif in red. (**E**) MDCK-II cells transiently transfected with GFP-coupled full-length NBCn1 (GFP-NCBn1), NBCn1 with deleted C-terminal (∆Ct-NBCn1), NBCn1 with a 4-amino acid deletion in the putative PDZ domain (ΔPDZ -NBCn1) or NBCn1 with mutated PDZ domain (EGSL-NBCn1). Fluorescence images of F-actin (magenta), GFP (green). Nuclei are stained with DAPI (blue). Scale bar 10 µm. Data are representative of 2–3 independent experiments per construct. Corresponding data for MCF-7 cells are shown in Fig. S2.

To investigate the mechanisms involved in NBCn1 membrane localization, we created a series of N-terminally GFP-tagged NBCn1 variants. In polarized MDCK-II cells, GFP-tagged full-length NBCn1 localized to the basolateral membrane in a manner undistinguishable from that of native NBCn1 (Fig. 2A). To verify that the exogenously expressed NBCn1 was functional, MCF-7 cells were transfected with the full-length GFP-NBCn1, and 48 h later, cells were loaded with the pH sensitive fluorophore 2′,7′-bis-(2-carboxyethyl)-5-(and-6)-carboxyfluorescein (BCECF) (Fig. 2B,C). Cells were subjected to NH_4_Cl prepulse-induced acidification in the presence of HCO_3_^−^/CO_2_, and recovery after acidification was monitored in GFP-NBCn1 transfected vs parallel untransfected controls. To separate the contribution of NBCn1 from the sizable endogenous Na^+^/H^+^ exchanger (NHE1) activity in these cells^4^, experiments were conducted in absence or presence of the NHE1 inhibitor 5′(*N*-ethyl-*N*-isopropyl)amiloride (EIPA). In presence of EIPA, the rate of recovery after acidification was significantly higher in GFP-NBCn1 transfected cells, compared to untransfected controls (Fig. 2B,C). Taken together, these results show that the GFP-tagged NBCn1 localizes to the plasma membrane and functions as a net acid extruder.

Next, we created N-terminally GFP-tagged, C-terminally deleted (∆Ct-NBCn1) NBCn1 and expressed it in MDCK-II cells (Fig. 2E,F) and MCF-7 cells (Fig. S2). Deletion of the C-terminal abolished plasma membrane localization of NBCn1 in both cell lines. Using the online MoDPepInt motif search engine (http://modpepint.informatik.uni-freiburg.de/PDZPepInt/Input.jsp) we confirmed that the last six C-terminal amino acids in NBCn1 (-DAETSL-COOH) constitute a putative PDZ-binding motif (Fig. 2E), consistent with previous reports^13^. Given the important role of PDZ-binding motifs in protein sorting and membrane retention^25^ we created a deletion variant missing the last four amino (ETSL) acids of this putative binding domain (ΔPDZ-NBCn1) and a variant harboring a single T → G mutation (E**G**SL-NBCn1). In contrast to the deletion of the entire C-terminal tail, deletion or mutation of the PDZ-binding motif did not detectably alter NBCn1 plasma membrane localization (Fig. 2F).

These results show that NBCn1 plasma membrane localization is dependent on the C-terminal tail but not on the PDZ-binding ETSL motif. This suggests that the NBCn1 C-terminal tail harbors motifs other than the putative PDZ-binding domain which are important for NBCn1 membrane trafficking and/or localization. It remains possible that the PDZ binding motif is important for trafficking of NBCn1 in other contexts, and its known interactions with other membrane proteins^13–16^ strongly suggests a role for this domain in NBCn1 organization at the plasma membrane.

### GST-pull-down and mass spectrometry reveal multiple NBCn1 C-terminal binding candidates relevant for sorting, trafficking and degradation

To gain further insight into the role(s) of the NBCn1 C-terminal in the membrane localization of the transporter, we cloned the NBCn1 C-terminal with a GST tag and carried out GST-pull-down with the NBCn1 C-terminal in MCF-7 cell lysates, followed by mass spectrometry (MS) analysis to identify possible binding partners. This revealed multiple possible interaction partners with a robust detection ratio over GST alone (Table 1).

**Table 1:** Putative binding candidates for NBCn1 C-terminal in MCF-7 cells.

Gene	Protein ID	**C-terminal Intensity 10** ^**6**^	**C-terminal/GST**
Protein sorting machinery			
AP2A1	AP-2 subunit alpha-1	11.0	1.3
AP2B1	AP-2 subunit beta	9.8	3.8
AP2M1	AP-2 subunit mu	16.8	4.4
CLTA	Clathrin light chain A	17.4	—
CLTC	Clathrin heavy chain 1	774.9	2.1
EEA1	Early endosome	1.3	2.6
Rab5A	Ras-related protein 5A	42.9	—
Rab5B	Ras-related protein 5B	10.4	—
Rab5C	Ras-related protein 5C	173.7	—
Rab8A	Ras-related protein 8A	40.0	—
Rab11	Ras-related protein 11	188.8	24.3
**Scaffolding proteins**			
GNB2L1	RACK1	205.0	—
CTTN	Cortactin	7.9	1.7
**Protein degradation machinery**			
LAMP1	Lysosomal membrane protein 1	118.9	22.9
LAMP2	Lysosomal membrane protein 2	35.8	20.6
UBA1	Ubiquitin activating enzyme 1 (E1)	801.9	3.7
UBA6	Ubiquitin activating enzyme 6 (E1)	343.0	46.9
UBE2V1	Ubiquitin conjugated enzyme (E2)	229.2	—
UBE2N	Ubiquitin conjugated enzyme (E2)	280.1	—
SYVN1	Ubiquitin ligase (E3)	1.6	—
UBR4	Ubiquitin ligase (E3)	26.3	7.6
RBX1	Ubiquitin ligase (E3)	21.5	—
HUWE1	Ubiquitin ligase (E3)	15.6	5.4
STUB1	Ubiquitin ligase (E3)	52.2	—
**Retromer and WASH complex**			
SNX1	SNX1	7.3	6.1
SNX2	SNX2	35.3	10.9
SNX5	SNX5	13.7	—
SNX6	SNX6	44.2	13.9
VPS26a	VPS26a	8.1	3.3
VPS26b	VPS26b	3.6	—
VPS29	VPS29	22.4	—
VPS35	VPS35	149.2	18.8
SNX27	SNX27	84.5	157.1
KIAA0196	Strumpellin	0.9	2.3
**Polarity complex**			
SCRIB	Scribble	2.2	—
LLGL1	Lethal giant larvae 1	0.96	—

Putative binding candidates for NBCn1 C-terminal in MCF-7 cells. The NBCn1-C-tail tagged with GST was produced recombinantly in *E. coli*. MCF-7 cells were grown to 90% confluency and lysed. NBCn1-C-GST-bound beads were added to the lysates, with GST-only conjugated beads and MCF-7 cell lysates with no beads as control conditions. Proteins were isolated and prepared for mass spectrometry, and data analysed, as described in Materials and Methods. An independent experiment conducted in MCF-7 cells expressing p95HER2 yielded similar results. The values listed in the table are intensities in millions (10^6^). A C-terminal/GST ratio was calculated based on intensities in the C-terminal pull-down and GST-pull-down. “–”, indicates no intensity measured for GST, thus no ratio calculated.

Several groups of proteins relevant for protein sorting, trafficking and degradation were identified (Table 1). Firstly, we found a large group of mediators involved in clathrin-dependent endocytosis, such as clathrin light and heavy chains, the adaptor protein (AP)2 complex subunits α-1, ß, and µ, and also endosomal Rab proteins such as Rab5A, -B, and -C as well as Rab11 and Rab8. A second group of identified proteins of interest were cortactin, which is important for membrane targeting of other plasma membrane proteins^30^, and the small scaffolding protein Receptor of activated protein C kinase 1 (GNB2L1, RACK1), which is involved in the trafficking, targeting and membrane retention of several of its binding partners^31^. A third group of identified putative binding partners comprised components of the protein degradation machinery, including Lamp-1 and -2, and several E1-, E2-, and E3- ubiquitin-conjugated enzymes, including at least five E3 ubiquitin ligases: SYVN1, UBR4, RBX1, HUWE1 and STUB1. Finally, we found a large fraction of the components of the retromer and WASH complex, which is involved in protein recycling from endosomes to the Trans-Golgi network or back to the cell surface, respectively^32^. Retromer components identified included sorting nexin SNX-1, -2, -5, -6 and vacuolar protein sorting VPS-26a/b, -29 and -35. Components from the WASH complex included SNX27 and Strumpellin (Table 1).

Taken together, the GST-pull-down and MS analysis revealed multiple novel NBCn1 C-terminal binding candidates of putative importance for the sorting, trafficking, membrane retention and degradation of NBCn1.

### RACK1 interacts with NBCn1 and is important for its plasma membrane localization

**Figure 3:**
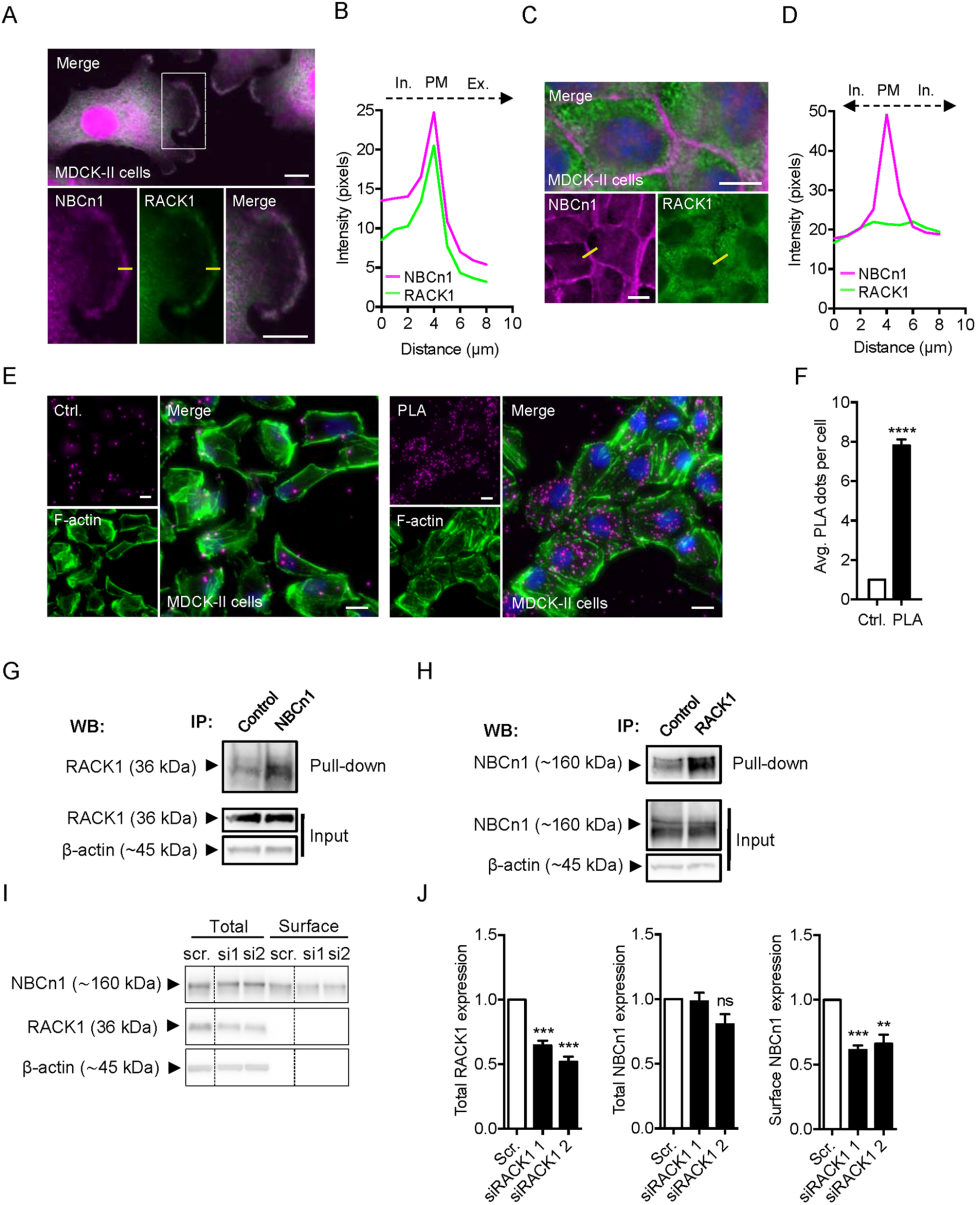
RACK1 interacts with NBCn1 and is important for NBCn1 membrane localization. (**A**) Fluorescence images of NBCn1 (magenta) and RACK1 (green) in non-confluent MDCK-II cells. Nuclei are stained with DAPI (blue). (**B**) Line scan of marked yellow line in A was carried out in ImageJ and captured across the membrane as illustrated. (**C**) Fluorescence images of NBCn1 (magenta) and RACK1 (green) in confluent MDCK-II cells. Nuclei stained with DAPI (blue). (**D**) Line scan of marked line in C was carried out in ImageJ and captured across the membrane as illustrated. Scale bar 10 µm. (**E,F**) Similar data were obtained in MCF-7 and SKBr-3 cells (Fig. S3A,B). Proximity ligase assay (PLA) carried out in MDCK-II cells. Cells were treated with primary antibodies directed against NBCn1 or RACK1 or only NBCn1 as a control. A positive PLA signal appears as magenta dots. F-actin was stained with phalloidin-488 (green). (**F**) quantification of PLA dots was carried out in ImageJ using the analyze particles application. PLA signal in 10 randomly chosen cells from 10 different image areas were counted, and the average PLA signal per cell is shown in the bar graph. Data are representative of 3 independent experiments and shown as mean with SEM error bars. ****P < 0.0001, Student’s *t* test. Similar data were obtained in MCF-7 cells (Fig. S3C,D). (**G,H**) Native co-IP of NBCn1 and RACK in non-confluent (~70% confluency) MDCK-II cells. Cells were lysed and subjected to pull-down with either antibody against NBCn1 (G) or against RACK1 (H), or the appropriate isotype control, followed by Western blotting of the pull-down and input fractions as shown. Representative of 4 independent experiments. (**I,J**) The surface fraction of NCBn1 in MCF-7 cells was examined by biotinylation after RACK1 knockdown using two different siRNAs. I: representative Western blots. ß-actin was used as a loading control. J: quantifications of RACK1 and NBCn1 protein expression normalized to mock control. **^,^***P < 0.01 and 0.001 vs. scrambled (Scr), respectively, ns: non-significant. n = 3.

A major interaction candidate revealed by the MS analysis was RACK1 (Table 1), which is known to play important roles in protein targeting and membrane retention^31,33–37^. Therefore, we hypothesized that RACK1 contributes to NBCn1 membrane targeting and/or retention. To test this, we first carried out immunofluorescence analysis of native NBCn1 and RACK1 to identify regions of co-localization of the two proteins. In non-confluent MDCK-II cells, NBCn1 and RACK1 both appeared to localize throughout the cell, and strong co-localization was evident particularly in leading edge lamellipodia (Fig. 3A,B). Remarkably, this distribution was altered in confluent MDCK-II cells: Under confluent conditions, NBCn1 was almost exclusively membrane-localized, while RACK1 exhibited a punctate, cytoplasmic localization (Fig. 3C,D). A similar pattern was observed in MCF-7 cells and in SKBr-3 breast cancer epithelial cells (Suppl. Fig. S3A,B), suggesting that this is a general phenomenon in epithelial cells.

Cellular interaction between NBCn1 and RACK1 was substantiated by *in situ* proximity ligase assay (PLA), an antibody-based assay that can detect close interaction (intermolecular distance < 40 nm) between two natively expressed proteins^38^. Demonstrating close proximity between the two proteins, non-confluent cells incubated with both NBCn1 and RACK1 primary antibodies showed a PLA signal about 8-fold above the control exposed to only NBCn1 antibody, both in MDCK-II (Fig. 3E,F) and MCF-7 cells (Fig. S3C,D). To determine whether this reflected physical interaction between the two proteins, we performed native co-immunoprecipitation (co-IP) between NBCn1 and RACK1 in confluent and non-confluent (70% confluency) MDCK-II cells (Fig. 3G,H). NBCn1 was able to precipitate RACK1 (Fig. 3G) and vice versa (Fig. 3H) in non-confluent cells, confirming their physical interaction. In some cases, however, Rack1 was able to precipitate NBCn1 also in confluent cells (data not shown). We finally asked whether RACK1 was important for the plasma membrane localization of NBCn1. A cell surface biotinylation assay was used to determine the surface fraction of NBCn1 following transfection of MCF-7 cells with two different siRNAs targeting RACK1 (Fig. 3I,J). RACK1 knockdown cells elicited a significant reduction of the NBCn1 surface fraction, comparable in magnitude to the reduction in RACK1 protein level, yet did not alter the total cellular NBCn1 protein level.

**Figure 4:**
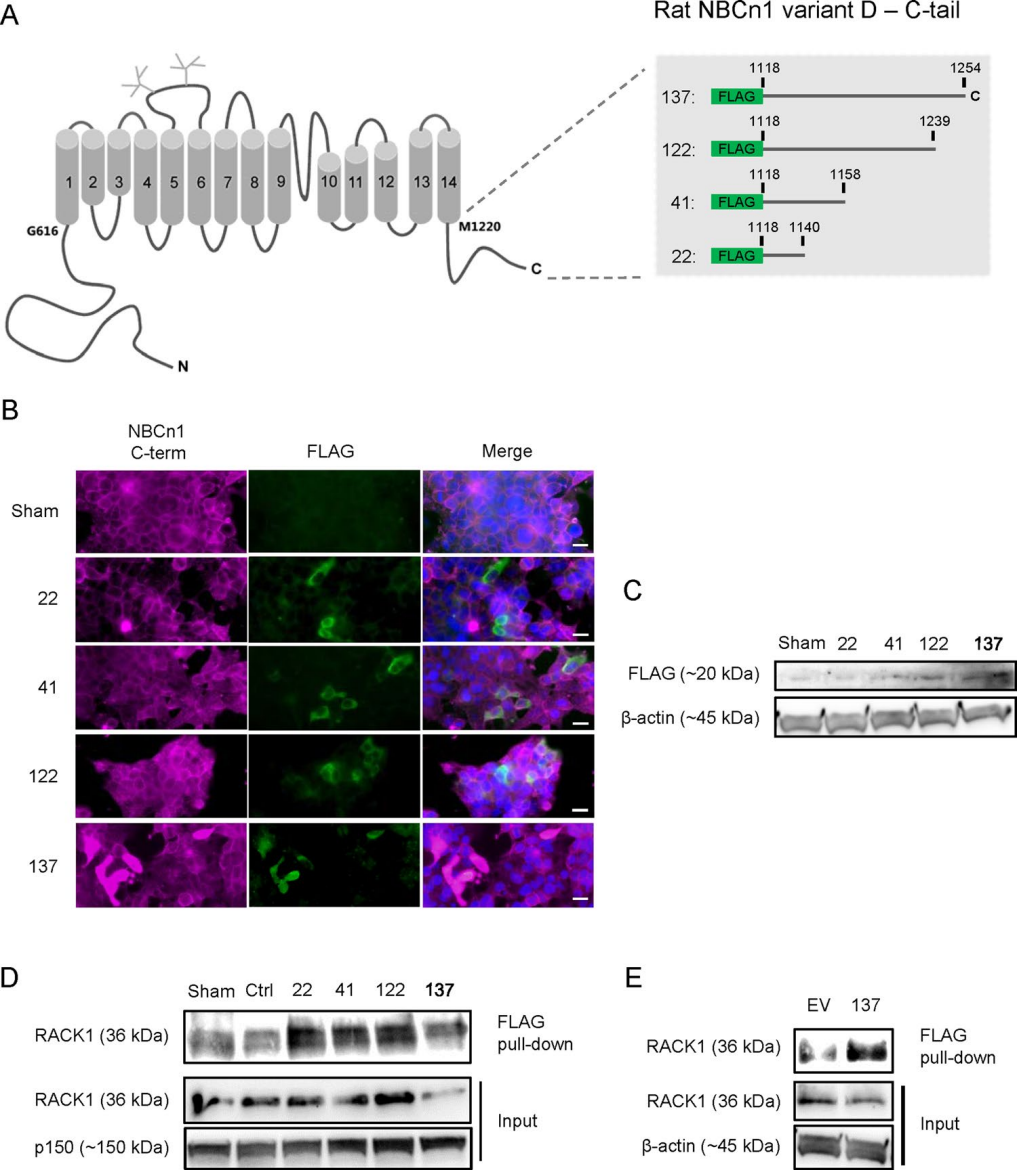
RACK1 interaction with FLAG-NBCn1 deletion constructs. (**A**) Overview of NBCn1 organization and the four FLAG-tagged NBCn1 constructs employed. HEK293 cells were transiently transfected with one full length (137) and three truncated (22, 41 and 122) FLAG-tagged versions of the NBCn1 C-terminal prior to lysis or PFA fixation after 24 h incubation. (**B**) Fixed cells were prepared for immunofluorescence microscopy using antibodies directed against the most distal C-terminal amino acids in NBCn1 (NBCn1 C-term) or FLAG. Scale bars: 20 µm. (**C**) Samples from lysed cells were subjected to SDS-PAGE and Western blotting against FLAG and β-actin (loading control). (**D**) Samples from lysed cells were subjected to FLAG IP and analyzed by SDS-PAGE and Western blotting against RACK1 and p150 (loading control). Ctrl: isotype control. Sham: sham-transfected cells. (**E**) HEK293 cells were transfected with empty vector pFLAG-CMV-2 (EV) or full length NCBn1 C-terminus (137) and lysed before performing IP against FLAG and analyzing by SDS-PAGE and Western blotting against RACK1 and β-actin (loading control). Representative images from 3–4 independent experiments are shown.

In order to determine which part of the NBCn1 C-tail interacted with RACK1, we designed a deletion series of FLAG-tagged C-tail constructs, spanning from the full C-tail (137 amino acids) to the most proximal 22 amino acids (Fig. 4A). The constructs were expressed in HEK-293 cells, as validated by immunofluorescence analysis and Western blotting for FLAG (Fig. 4B,C). Lysates from the transfected cells were subjected to FLAG co-IP followed by blotting for RACK1. As seen, all four constructs pulled down RACK1 to a greater extent than that seen with control transfections (sham, IgG ctrl., and empty vector) (Fig. 4D,E).

Collectively, these results show that RACK1 physically interacts with NBCn1, that the most proximal part of the NBCn1 C-tail is sufficient for this interaction, and that NBCn1 and RACK1 co-localize most prominently in non-confluent cells. Furthermore, RACK1 knockdown is associated with reduced NBCn1 surface expression, but not with changes in its total expression level.

### NBCn1 undergoes rapid constitutive internalization in MDCK-II cells

**Figure 5:**
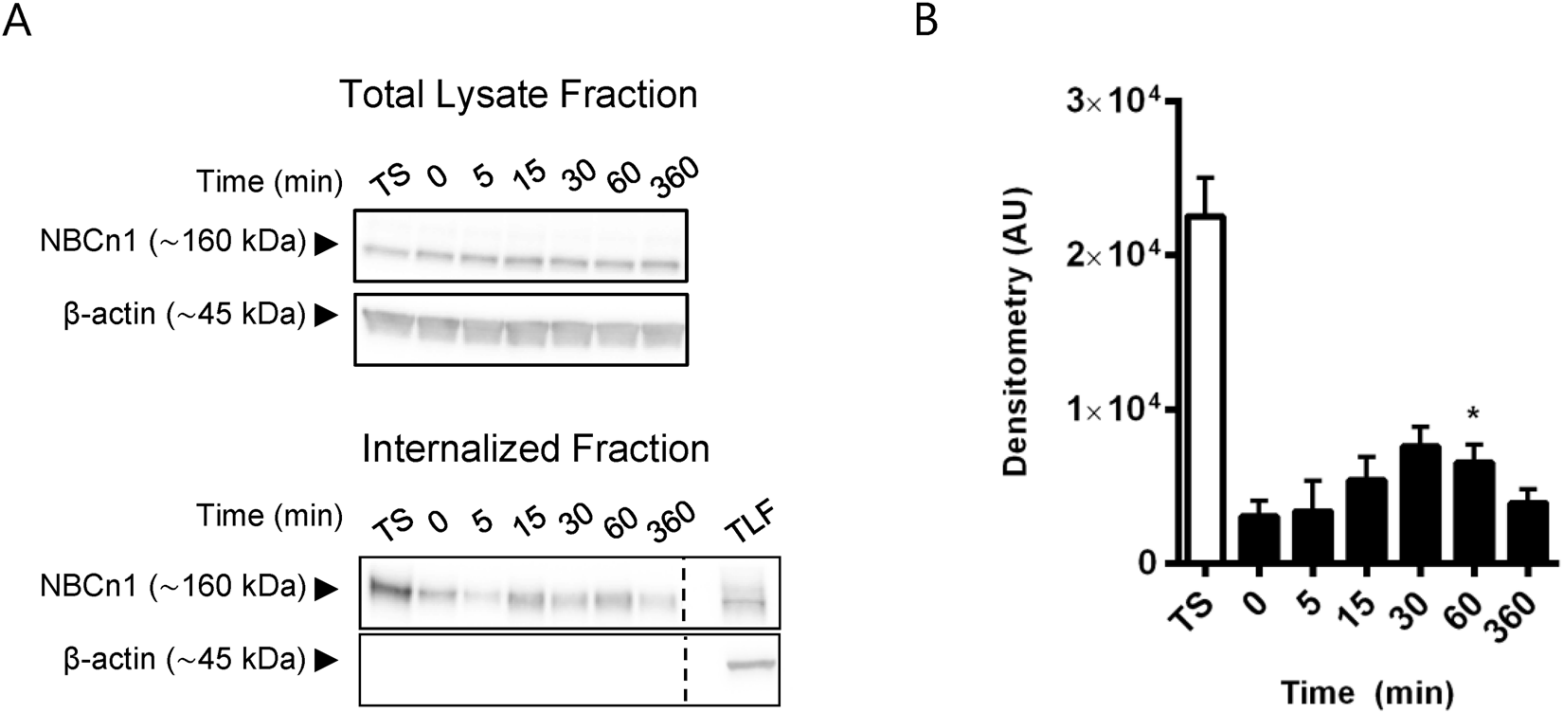
NBCn1 is rapidly endocytosed from the basolateral membrane. MDCK-II cells were cultured on Transwell filters for 4 days and basolateral surface expressed proteins were labeled with cell impermeable biotin at 4 °C. Internalization was carried out at 37  °C, followed by stripping of biotinylated proteins still exposed at the cell surface. Cells were lysed and analyzed by Western blotting at the times indicated. (**A**) Representative Western blots of the total lysate fraction (top) and internalized (bottom) NBCn1. ß-actin was used as a loading control. TS: Total surface expression, TLF: total lysate fraction. For the 0 min time point, stripping off surface biotin was performed immediately after biotinylation. (**B**) Internalized NBCn1. Quantification of C showing mean raw densitometry (+SEM) of three independent experiments in arbitrary units (AU). *p < 0.05 One-way ANOVA with Dunnett’s post-hoc test (0 min is control column).

The results from the MS analysis suggested that the NBCn1 C-terminal region interacts with multiple proteins involved in endocytosis, including several components of the clathrin-mediated endocytosis pathway, such as the AP-2 complex, clathrin itself and Rab5 (Table 1). We therefore next asked whether NBCn1 is constitutively internalized in polarized MDCK-II cells. Cells were grown on Transwell filters for 4 days to allow polarization. Proteins accessible from the basolateral cell surface were labeled with cell-impermeable biotin at 4 °C. Cells were subsequently incubated at 37 °C under standard cell culture conditions to allow internalization for 0 (control), 5, 15, 30, 60 and 360 min. Total surface biotin labeled proteins were detectable only at the (baso)lateral membrane confirming that biotinylation was specifically basolateral (n = 3, data not shown). Subsequent to internalization, remaining biotin was stripped from surface proteins to allow specific tracing of internalized protein, and samples were subjected to streptavidin pulldown to isolate biotinylated proteins, corresponding to internalized proteins. To evaluate internalized NBCn1 at the different time points, pulldown samples (internalized proteins) were subjected to Western blotting against NBCn1. Following 15 min of internalization at 37 °C, an increased level of internalized NBCn1 protein was detectable by Western blotting, demonstrating that NBCn1 is internalized from the basolateral membrane already at this time (Fig. 5A,B). NBCn1 internalization reached peak levels around 30–60 min after transfer to 37 °C. This was followed by a reduction in detectable biotinylated NBCn1, reaching approximately the 0 min level at time point 360 min, and presumably reflecting protein degradation.

These results show that NBCn1 undergoes rapid constitutive endocytosis in polarized MDCK-II epithelial cells.

### Total cellular NBCn1 turnover is slow and dependent on lysosomal function

**Figure 6:**
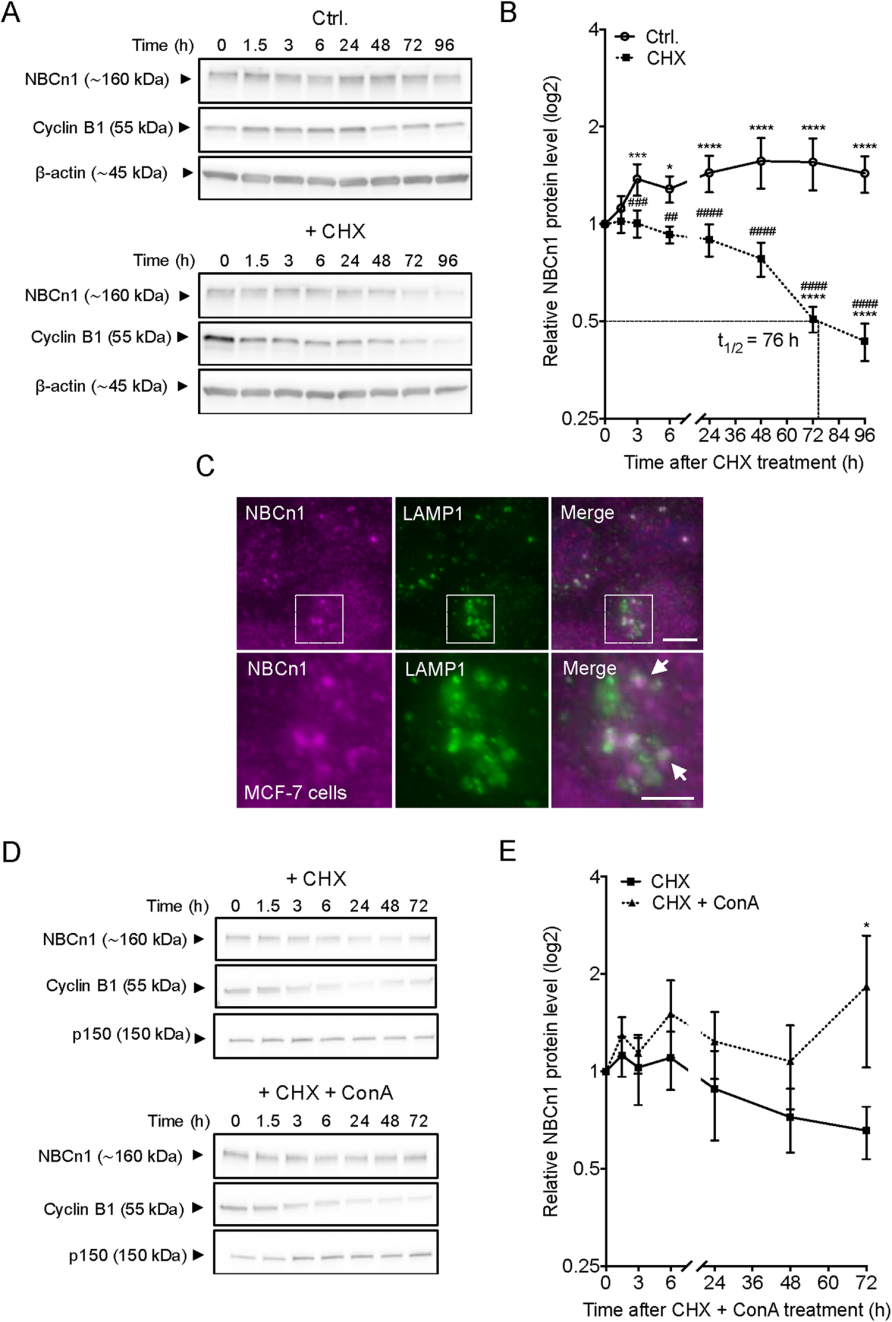
Protein turnover of NBCn1 is very slow. MCF-7 cells were cultured for 48 h prior to exposure to 100 µg/ml cycloheximide (CHX)+/− 10 nM concanamycin A (ConA) for the indicated time. Cells were lysed and processed for Western blotting (**A**,**B** and **D**,**E**) or fixed for immunofluorescence analysis (C). (**A**,**D**) Representative Western blots. ß-actin was used as a loading control and Cyclin B1 was used as a positive control for protein synthesis inhibition. (**B**,**E**) Protein turnover rate for NBCn1 after exposure to CHX+/− ConA. Values are normalized to 0 h for each exposure. (**C**) Fluorescence images of NBCn1 (magenta) and LAMP1 (green). Nuclei are stained with DAPI (blue). Scale bar 5 µm. Data are representative of 3–5 independent experiments. Western blot data are shown as mean with SEM error bars. *: comparison of values under the same treatment; #: comparison of values at the same condition but subjected to different exposures. */#, **/##, ***/### and ****/#### indicate P < 0.05, P < 0.01, P < 0.001 and P < 0.0001 respectively, relative to the initial value for that group (stars) or to the corresponding non-CHX-treated controls at the same time point (hash-tags). Two-way ANOVA with Bonferroni post-test. Similar data were obtained in MDCK-II cells (Fig. S5).

Our recent findings indicated that NBCn1 protein turnover is slow in MCF-7 cells^9^, however, the exact half-life of the transporter and the mechanism of degradation were not determined. To address this, MCF-7 cells were exposed to 100 µg/ml cycloheximide (CHX) to arrest protein synthesis, and NBCn1 protein levels were determined at 8 time points from 0–96 h after CHX addition (Fig. 6A,B). In cells not exposed to CHX (solid line, Fig. 6B), the NBCn1 protein level was significantly increased after 6 h and continued to increase for the first 48 h. Cyclin B1 was used as a positive control due to its rapid protein turnover^39^. As expected, Cyclin B1 was rapidly degraded with an estimated half-life of less than 3 h. In contrast, NBCn1 degradation was much slower, with an estimated half-life of 76 h (Fig. 6B). NBCn1 degradation was also slower than that of NHE1, another pH-regulatory membrane protein, the half-life of which has previously been estimated to 24–48 h (Fig. S4A,B)^40^. At time points later than 48 h, CHX treatment elicited modest but detectable poly ADP-ribose polymerase (PARP) cleavage (Fig. S4C). However, this is unlikely to affect NBCn1 expression data, which was always normalized to total protein expression and β-actin loading controls.

We previously reported that the proteasome inhibitor MG132 did not affect degradation of total cellular NBCn1, arguing against a detectable contribution from proteasomal degradation to NBCn1 turnover^10^. We therefore hypothesized that NBCn1 is primarily degraded in the lysosomal compartment. Co-immunofluorescence analysis of NBCn1 and the lysosomal membrane protein LAMP1 revealed extensive co-localization between NBCn1 and LAMP1 in MCF-7 cells (Fig. 6C). Further, co-treatment with concanamycin A (which inhibits the lysosomal H^+^ V-ATPase and hence lysosomal function) over a period of 0–96 h blocked the loss of NBCn1 protein after CHX treatment (Fig. 6D,E). Similar findings were obtained in MDCK-II cells (Fig. S5), supporting the generality of this mechanism.

These results show that NBCn1 turnover proceeds with a half-life of approximately 76 h, and suggest that NBCn1 is predominantly degraded in the lysosomes.

## Discussion

Despite the well described physiological roles of NBCn1^1,2^ and the fact that dysregulation of NBCn1 contributes to the development of several human malignancies, including breast cancer^1,7,8^, the kinetics and mechanisms of sorting, recycling, localization and degradation of NBCn1 are largely unexplored. This work provides the first analysis of NBCn1 targeting, sorting and degradation in epithelial cells.

In renal medulla, duodenum and exocrine gland epithelial cell types^2,22,27,41^, with the exception of a small part of the choroid plexus epithelium^42^, NBCn1 localizes to the basolateral membrane. As expected, this was also the case in polarized MCF-7 and MDCK-II cells. Deletion of the NBCn1 C-terminal abolished the plasma membrane localization of NBCn1, confirming findings by Loiselle and co-workers^24^. We note, however, that NBCn1 is found predominantly in the lateral compartment rather than evenly distributed throughout the basolateral membrane and our MS analyses suggest multiple lateral interaction partners (e.g. Scribble and Lethal giant larvae 1).

A key finding of this study is that NBCn1 interacts with the scaffold protein RACK1, a finding supported by *in situ* PLA, reciprocal native co-IP, and pulldown of RACK1 with FLAG-tagged NBCn1 C-tail constructs. Although the precise minimal interaction motif was not identified, the most proximal part of the NBCn1 C-tail was sufficient for the interaction with RACK1. RACK1 interaction motifs are diverse and many interactions occur with multiple WD repeats and involve either a relatively small, specific motif, or a complex interaction over an extended surface^43^. In this context it is notable that the proximal part of the NBCn1 tail is predicted to be helical in nature (https://predictprotein.org/), and the RACK1-interaction domain on the cyclic AMP specific phosphodiesterase, PDE4D5, was proposed to be an amphipatic helix^44^. RACK1 knockdown reduced the cell surface expression of NBCn1, yet not its total cellular expression. This is in congruence with several studies demonstrating the importance of RACK1 for the surface expression of other membrane transporters and receptors^34–37,45^. Thus, the NBCn1-RACK1 interaction is important for NBCn1 surface expression and hence for the cellular capacity for net acid extrusion. Importantly, the marked co-localization of RACK1 and NBCn1 observed in motile, non-confluent MDCK-II cells was abolished (or undetectable) in fully polarized cells, indicating that the complex of proteins interacting with NBCn1 is dynamic and differs between stable polarized epithelial sheets and cells with a more mesenchymal phenotype. In congruence with our observations, the Thromboxane A2 receptor and RACK1 localized predominantly to the plasma membrane and in cytoplasmic puncta, respectively^36^, yet RACK1 played an important role in the membrane trafficking of the receptor, by a mechanism proposed to involve a transient interaction with RACK1 in the ER^36^. Thus, we suggest that NBCn1 trafficking to its lateral localization during apico-basal polarization of epithelial cells involves its RACK1-dependent trafficking from the ER to the leading edge, followed by formation of a new NBCn1 complex at the membrane which does not involve RACK1. In agreement with such a model for the function of RACK1, the transmembrane protease ADAM12 localized mainly intracellularly in subconfluent cultures, and under these conditions, RACK1 was important for its PKC-dependent membrane translocation^45^. Finally, the BK_Ca_ channel was shown to interact with RACK1 by co-IP and GST-pulldown, but only a fraction of the channels and RACK1 co-localized in the plasma membrane^46^. While not studied here, the role of RACK1 in trafficking of other membrane proteins to the plasma membrane has been proposed to involve PKC-^36,45^ and dynamin-^36^ dependent mechanisms.

An unexpected finding of the present work, confirmed in both MCF-7 and MDCK-II cells, was that while the NBCn1 a space was missing here C-terminal tail was required (consistent with previous work,^24^), the PDZ binding domain at the distal end of the C-terminal was not required for membrane targeting of the transporter. This domain may nonetheless play a role in transporter recycling, since another prominent binding partner identified here was the retromer trafficking protein sorting nexin 27 (SNX27), which was recently shown to interact with NBCn1 in a PDZ-dependent manner^47^. Candidate regulators of basolateral sorting of NBCn1 identified in our MS analysis include the small GTPase Rab8 and Rab11. Several putative membrane sorting motifs are found in the NBCn1 C-terminal and ongoing work in our laboratory addresses the specific motifs and interactors involved in basolateral NBCn1 sorting and recycling.

By following the internalization of the pool of biotin labeled surface NBCn1 protein in MDCK-II cells we found that NBCn1 was rapidly internalized to a detectable level already after 15 min. After 360 min, the level of internalized NBCn1 was similar to control levels (0 min internalization). The rapid internalization and long half-life could reflect a fast and continuous NBCn1 recycling but further studies are needed to elaborate this speculation. A fast internalization rate is also seen for many other membrane transporters, including the Na^+^-Cl^−^ co-transporter, NCC (SLC12A3)^48^ and the Na^+^-H^+^ exchanger NHE3 (SLC9A3)^49^. Both undergo rapid clathrin-dependent endocytosis from the apical membrane as part of the dynamic regulation of their activity at the plasma membrane, whereas constitutive internalization of the basolateral NHE1 is substantially slower, with only 15% internalized after 3 h^50^. In contrast to our findings, a lack of detectable NBCn1 internalization was reported for NBCn1 in rat parotid gland acinus-derived ParC5 cells, while the electrogenic NBCe1 (SLC4A4), which shares 50% sequence similarity with NBCn1, was found to undergo clathrin-mediated endocytosis within 60 min^27^. It seems reasonable to speculate that the major differences between acinar cells and ductal epithelial cells may underlie the apparent contrast in endocytic properties, and this may suggest important differences in NBCn1 regulation in the two settings.

Using CHX to block *de novo* protein synthesis, we determined the half-life of NBCn1 protein degradation in MCF-7 cells to be 76 h. This confirms our recent finding that NBCn1 protein degradation is very slow^10^. The co-localization of NBCn1 with the lysosomal marker Lamp-1, and the fact that inhibition of the lysosomal V-ATPase inhibited NBCn1 degradation, suggests that NBCn1 degradation is predominantly lysosomal. This is in congruence with our previous finding that proteasomal inhibition has no effect on NBCn1 protein degradation^10^ and fits well with the detection of NBCn1 in the lysosomal proteome^51^. It is also consistent with the observation that lysosomal NBCn1 abundance was increased upon expression of p95HER2^51^, which is associated with greatly increased NBCn1 expression^4,9^. It remains possible that specific signaling pathways additionally stimulate NBCn1 internalization under certain regulatory conditions. Supporting this notion, we recently showed that upon cisplatin-induced cell death in MCF-7 breast cancer cells, NCBn1 was lost from the plasma membrane and appeared in a perinuclear compartment within 18 h, whereas NHE1 remained at the cell surface^28^. Five lysines in the human NBCn1 C-terminal, K1144, K1151, K1183, K1186, and K1206, have been shown to be ubiquitinated^52^. Consistent with this, our MS analysis identified several E3 ubiquitin ligases: SYVN1, UBR4, RBX1, HUWE1, and STUB1 (CHIP), as putative NBCn1 C-terminal interaction partners. At least four of these E3 ligases have been assigned roles in the degradation of ion transport proteins^53–56^, and could be relevant candidates for NBCn1 ubiquitination. It thus seems likely that ubiquitination is a means to regulate NBCn1 internalization and degradation, as seen e.g. for the epithelial Na^+^ channel ENaC and the Na^+^/H^+^ exchanger NHE1^50,57^. Given the robust upregulation of NBCn1 in some epithelial cancer cells^6–8^, it is also interesting in this regard that one of the identified E3 ligases is STUB1, a tumor suppressor protein in several human cancers^58^. Clathrin and the AP2 clathrin-adaptor complex were also identified as possible NBCn1 C-terminal binding partners in our MS analysis, and may contribute to the ubiquitination-dependent internalization and lysosomal targeting of NBCn1^59^. The regions of potential interaction with AP2 were not studied here, but the NBCn1 C-terminal tail contains two classical di-hydrophobic such motifs (^1165^DDTVHL^1170^ and ^1173^EGGSLL^1178^), as well as two long basic stretches ^1142^KKKK^1145^ and ^1149^KKKK^1152^ which could act as non-canonical AP2 interaction motifs^60,61^.

In conclusion, here we identified a series of potential C-terminal NBCn1 binding partners, of which we demonstrated that the scaffold protein RACK1 interacts with NBCn1 and is important for its surface expression and hence net acid extrusion activity. While the NBCn1 C-terminal tail is required for its membrane localization, the PDZ-binding motif in its most distal end is dispensable for this process. Furthermore, we show that NBCn1 undergoes rapid constitutive endocytosis. In contrast, total NBCn1 protein turnover is very slow, with a half-life of about 76 h in MCF-7 epithelial cells, suggesting extensive recycling of the transporter protein. This work reveals novel aspects of the sorting, targeting, and degradation of NBCn1 and can contribute to a broader understanding of how NBCn1 is regulated in epithelial cells under normal and pathological conditions.

## Materials and Methods

### Antibodies and reagents

The polyclonal antibody directed against the N-terminal of NBCn1 was kindly provided by Jeppe Prætorius, Aarhus University, Denmark. Antibodies against ZO-1 (sc-10804), and GFP (sc-8334) were from Santa Cruz Biotechnology, Texas, USA. Antibodies directed against RACK1 (#610177 and #A2560) and E-cadherin (#610181) were from BD Transduction Laboratories, San Jose, USA and ABclonal, Woburn, USA, antibody against Cyclin B1 (#12231) was from Cell Signaling Technology, Danvers, USA, and antibodies against biotin (#B3640) and ß-actin (#A5441) and FLAG M2 (#F1804) were from Sigma-Aldrich, St. Louis, USA. Cycloheximide (#C7698) and Concanamycin A (#C9705), both from Sigma-Aldrich, were dissolved at 5 mg/mL in ddH_2_O and 10 µM in DMSO, respectively.

### Cell lines and culture conditions

MCF-7 cells were cultured in DMEM 1885 supplemented with 5% fetal bovine serum (FBS), 1% penicillin/streptomycin and 1% non-essential amino acids. MDCK-II cells were cultured in alpha modified MEM (α-MEM) supplemented with 10% FBS, 1% penicillin/streptomycin and 2 mM L-Glutamine. SKBr-3 and HEK293 cells were cultured in DMEM supplemented with 10% FBS and 1% penicillin/streptomycin. All cell lines were maintained at 37 °C, 95% humidity and 5% CO_2_. MCF-7 and MDCK-II cells were grown in culture dishes or, where indicated, grown for 4 days on Transwell polycarbonate filters (Corning Incorporated (Costar), Sigma-Aldrich, #3401 and #3412) with 0.4 µm pore size.

### GFP-tagged NBCn1 constructs

#### GFP-constructs

Rat NBCn1 (rNBCn1-D, NM_058211.2) was amplified by PCR from cDNA in plasmid kindly provided by Dr. Ebbe Boedtkjer (Aarhus University, Denmark). Amplified rNBCn1-D was inserted into a pEGFP-C1/Kan/Neo vector using the restriction enzymes BglII and KpnI (recognition sites shown in bold non-capital letters in the primers) (New England Biolabs) to produce the N-terminally GFP-tagged rNBCn1-D. Four different variations were made: Full-length NBCn1 (GFP-NBCn1); NBCn1 with the four C-terminal amino acids of the PDZ motif (ETSL) deleted (ΔPDZ-NBCn1); NBCn1 with a mutated PDZ motif (EGSL-NBCn1); and NBCn1 lacking the entire C-terminal tail (∆C-NBCn1). Primers used were: FW (All Constructs): 5′GATA**agatct**ATGGAGGCAGACGGGGCCGG′3; RV (GFP-NBCn1): 5′AATGGTACC**ggtacc**TTACAATGAAGTTTCAGCATCCATGTATTTTTTTG′3; RV (ΔPDZ-NBCn1): 5′AATGGTACC**ggtacc**TTAGCGCACAAAGACTAATGCAAGAACC′3; RV (EGSL-NBCn1): 5′AATGGTACC**ggtacc**AGCATCCATGTATTTTTTTGATGGTTCATC′3; RV (ΔCt-NBCn1): 5′AATGGTACC**ggtacc**CAATGAACCTTCAGCATCCATGTATTT′3.

Plasmids were transiently expressed in MDCK-II or MCF-7 cells using lipofectamine 3000, according to the manufacturer’s instructions. The following day, cells were fixed and subjected to immunofluorescence analysis as described below.

### Analysis of NBCn1 half-life

To estimate protein half-life of NBCn1, 100 µg/ml cycloheximide (CHX) and/or10 nM Concanamycin A (ConA) was added 48 h after seeding of MDCK-II or MCF-7 cells in Petri dishes. Cells were subsequently incubated for the indicated amount of time before they were lysed and analyzed by Western blotting as described below.

### Immunofluorescence analysis

Cells were washed in cold phosphate-buffered saline (PBS; 137 mM NaCl, 2.7 mM KCl, 8.1 mM Na_2_HPO_4_-2H_2_O and 1.5 mM KH_2_PO_4_), fixed for 30 min at 4 °C in 4% paraformaldehyde (PFA, Sigma-Aldrich, #47608) and washed in PBS. Cells were permeabilized in 0.5% Triton-X-100 (Plusone, #17-1315-01) for 10 min and blocked in 5% Bovine Serum Albumin (BSA, Sigma Aldrich #A7906) for 30 min. Primary antibodies were added over night at 4 °C and secondary antibodies for 1 h at room temperature. Cells were treated with DAPI (Invitrogen, Waltham, USA, #C10595) for 5 min to stain nuclei, washed in 1% BSA and mounted in 2% N-propyl-galleate (Sigma-Aldrich, #P-3130) in PBS/glycerine. Images were collected on an Olympus BX63 upright microscope with a 60 ×/1.35 NA oil objective using Olympus CellSens dimension software or (confocal images) on a Leica SP 5 × MP confocal laser scanning microscope with a 40 × or 60 ×/1.3 NA oil objective using Leica LAS AF software. Z-stacks were captured in intervals of 0.2 µm with corresponding Z-scans. Images were processed in ImageJ. No labeling was detectable in the absence of primary antibody at the settings used (data not shown).

### Cell surface biotinylation assay

To determine the surface fraction of NBCn1, MDCK-II cells were cultured on Transwell filters for 4 days in order to allow polarization. Cells were incubated for 30 min at 4 °C with freshly made 0.2 mg/ml cell impermeable EZ-Link Sulfo-NHS-SS-Biotin (Life Technologies, #21331) diluted in PBS added to either the basolateral or apical side of the filters as indicated. Removal of excess biotin was done by several washing steps in cold quenching buffer (0.1 M glycine in PBS). Cells were lysed in cold RIPA buffer (150 mM NaCl, 50 mM Tris HCl pH 7.5, 0.1% SDS, 0.5% Sodium deoxycholate, 1% Igepal CA630 and Complete^TM^ mini protease inhibitor). Samples were centrifuged for 15 min at 16.000 RCF and 4 °C, and the supernatant was adjusted to equal amounts of protein (DC assay, BioRad). A small fraction of the supernatant was dissolved in 4 × LDS sample buffer and saved as the Total Lysate Fraction (TLF). The remaining supernatant was incubated with prewashed streptavidine-conjugated agarose beads (Sigma-Aldrich, #S1638) for 2 h with gently rolling at 4 °C. Samples were washed several times in cold RIPA buffer, dissolved in 2 × LDS sample buffer and heated for 5 min at 95 °C. Beads were pelleted by centrifugation at 4 °C for 4 min at 2000 RCF and the supernatant was saved as the Pull-down (PD) fraction. Samples were processed for Western blotting as below.

### SDS-PAGE and Western blotting

Cells were lysed in 95 °C pre-heated SDS lysis buffer (0.1 M Tris-HCl pH 7.5, 10% SDS, 1 mM Na_4_VO_3_ and Complete^TM^ protease inhibitor mix (Roche)). Protein concentrations were determined by DC assay (BioRad, Hercules, USA) and samples were mixed with NuPage LDS sample buffer (Invitrogen, #NP0007). Equal amounts of protein were separated by SDS-PAGE using Criterion^TM^ 10% tris gels (Bio-Rad, 18 wells, #567-1034 and 26 wells, #567-1035) and premade Tris/Glycine/SDS running buffer (BioRad, #161-0732), and BenchMark protein ladder (Invitrogen, #10747-012). Proteins were transferred by Trans-Blot Turbo transfer system (BioRad) to a PVDF membrane (BioRad, #170-4159), stained with Ponceau S and blocked in 5% nonfat dry milk in TRIS-buffered saline with Tween (TBS-T; 0.01 M Tris/HCL, 0.15 M NaCl, 0.1% Tween 20, pH 7.4) for 1 h at 37 °C. Membranes were incubated with primary antibodies diluted in 5% nonfat dry milk in TBS-T overnight at 4 °C and with horseradish peroxidase (HRP) conjugated secondary antibodies in 5% nonfat dry milk in TBS-T for 1 h at room temperature. Bands were visualized by chemiluminescent using enhanced chemiluminescent (ECL) substrate (Pierce, #32106). Densitometric analyses were carried out using ImageJ.

### Glutathione-S-Transferase (GST)-pulldown and mass spectrometry analysis

The NBCn1 C-terminal was amplified from a pcDNA5 plasmid containing full length rat NBCn1-D using the primers: FW: 5′TA**ggatcc**GTGGCGAGGCTCATGGAT′3 and RV: 5′GACG**gaattc**TTACAATGAAGTTTCAGC′3) and inserted with BamHI (FW) and EcoRI (RV) restriction enzymes (shown in bold non-capital letters) at the C-terminal of the GST gene under the control of a lactose operon in a pGEX-2T vector containing. The plasmid was expressed in *E. coli*, and protein synthesis of the NBCn1 C-terminal-GST (NBCn1-C-GST) construct was initiated by adding 1 mM isopropyl ß-D-thio-galactoside (IPTG, Sigma-Aldrich, #11284) followed by overnight incubation at 4 °C under gently shaking. IPTG-induced bacteria were spun down for 13 min at 2800 RCF and the pellet re-suspended in cold PBS supplemented with protease inhibitor mix (Roche). Samples were sonicated and centrifuged for 20 min at 20.000 RCF and added to a Bio-rad poly-prep chromatography column (Bio-Rad, #731–1550) containing glutathione agarose beads (Life Technologies, Waltham, USA, #G2879) washed in PBS supplemented with 0.05% Triton-X-100. The NBCn1-C-GST-bound beads were collected and stored in 100% glycerol at −80 °C. MCF-7 cells were cultured to 90% confluency, lysed in cold PBS, trypsinized and centrifuged at 50 RCF. Pellet were re-suspended in cold GST-lysisbuffer (80 mM Tris-HCl pH 7.5, 200 mM NaCl, 1% Triton-X-100 and protease inhibitor mix (Roche)), sonicated and protein concentration was measured by DC assay (Bio-Rad). Washed NBCn1-C-GST-bound beads were added to the lysates. GST-only conjugated beads and MCF-7 cell lysates with no beads were used as controls. The bead-lysate mixes were incubated for 2 h at 4 °C with gentle rolling, washed several times in wash buffer (20 mM Tris HCl pH 7.5, 150 mM NaCl, 1 mM DTT and 0.05% Triton-X-100), and adjusted to 10 µg/µl with wash buffer. To prepare samples for mass spectrometry, proteins were separated on Bis-TRIS gels followed by Coomassie staining, excision, protein digestion with trypsin and protein desalting to avoid interference of ions with the mass spectrometry analysis. The digested protein fragments were analyzed by HPLC coupled LTQ Orbitrap mass spectrometry^62^. Mass spectrometric analysis was performed by LC-MS using an Easy-LC system (Thermo) and an LTQOrbitrap or Q-Exactive mass spectrometer (Thermo Fisher). The raw data files were processed using MaxQuant (version 1.3.05)^63^. Data were blasted against Uniprot (http://www.uniprot.org/) human proteome dataset (release 2013_01, 87613 sequences) using the Andromeda search engine^64^.

### *In situ* proximity ligation assay (PLA)

Proximity between NBCn1 and RACK1 was detected using the Duolink II Detection Reagents Red kit (Sigma Aldrich, #DUO92004) according to the Manufacturer’s instructions. Briefly, MCF-7 and MDCK-II cells were seeded on coverslips 48 h prior to assaying to allow 60–70% confluency. Cells were washed in cold PBS, fixed for 30 min in 4% PFA on ice, washed in Duolink II Buffer A, quenched for 15 min in 0.1 M glycine in PBS, permeabilized in 0.5% Triton-X-100 and washed in Buffer A. Cells were blocked in O-link blocking solution for 30 min, incubated with primary antibodies for 1 h and with PLA probes diluted 1:5 for 1 h, both at 37 °C. Cells were washed in Buffer A and incubated with ligation solution for 30 min, and amplification solution for 100 min, both at 37 °C. Preparations were incubated with Alexa-488-conjugated phalloidin (Invitrogen, #A12379) for 1 h to stain F-actin, and were finally washed in Buffer A, treated with DAPI for 5 min to stain nuclei, washed in ddH_2_O and mounted using 2% N-propyl-galleate in PBS/glycerine mounting medium. Images were collected on an Olympus BX63 upright microscope with a 60 ×/1.35 NA oil objective using Olympus CellSens dimension software. Z-stacks were captured in intervals of 0.7 µm and processed in ImageJ. Quantification of the PLA dots were carried out in ImageJ by using ROI manage and the “Analyze particles” functions.

### Live imaging of intracellular pH

MCF-7 cells were seeded in WillCo glass bottom dishes (WillCo Wells, Amsterdam, The Netherlands, #3522) and transfected with full-length GFP-NBCn1-D or left untransfected (control cells). Cells were incubated in growth medium containing 2.5 µM 2′,7′-bis-(2-carboxyethyl)-5-(and-6)-carboxyfluorescein acetoxymethyl ester (BCECF-AM Invitrogen #B1150) for 30 min at 37 °C, followed by transfer to HCO_3_^−^ containing Ringer solution (118 mM NaCl, 25 mM NaHCO_3_, 5 mM KCl, 1 mM MgSO_4_, 1 mM Na_2_HPO_4_, 1 mM CaCl_2_, 3.3 mM MOPS, 3.3 mM TES, 5 mM HEPES, adjusted with NaOH to pH 7.4 at 37 °C). GFP fluorescence was negligible compared to the BCECF fluorescence, and did not interfere with pH_i_ measurements. The pH_i_ recovery was assessed by using the NH_4_^+^-prepulse technique: after a 5 min baseline phase, cells were exposed to 20 mM NH_4_Cl in the same NaHCO_3_ containing saline, for 10 min. Removal of NH_4_Cl leads to a rapid acidification, the initial slope of recovery from which is the rate of net acid extrusion. Recovery was assessed in absence or presence of 10 µM EIPA to inhibit Na^+^/H^+^ exchange. pH_i_ was measured using a Nikon Eclipse Ti microscope equipped with a Xenon lamp, a 40 × oil, 1.4 NA objective and EasyRatioPro imaging software (PTI, NJ,USA). The excitation wavelengths alternated between 440 nm and 485 nm, and emission was measured at 520 nm. The BCECF ratio was calculated from the fluorescence intensities measured at 485 nm/440 nm after correction for background fluorescence. Calibration was carried out using the high K +/nigericin technique^65^, employing KCl solutions (156 mM KCl, 1 mM MgSO_4_, 1 mM CaCl_2_, 1 mM K_2_HPO_4_, 3.3 mM 3-(-N-morpholino)propanesulfonic acid (MOPS), 3.3 mM 2-[Tris(hydroxymethyl)-methylamino]-ethanesulfonic acid (TES), 5 mM 4-(2-hydroxyethyl)-1-piperazineethanesulfonic acid (HEPES)) of pH 6.6, 7.0, 7.4 and 7.8, and 5 µM Nigericin (Sigma-Aldrich, Cat.# N-7143).

### Native co-immunoprecipitation of NBCn1 and RACK1

MDCK-II cells were seeded in 10 cm Petri dishes, washed twice in PBS and lysed with room temperature lysis buffer (50 mM Tris pH 7.4, 140 mM NaCl, 3 mM Na_3_VO_4_, 1% v/v IGEPAL CA-630 (Sigma, cat. #I8896), Phosphatase Inhibitor cocktail (PhosStop) (Roche, cat. #04906845001), Protease Inhibitor cocktail (Roche, cat. #11697498001). Protein content was normalized to a concentration of 3–4 mg/mL in 500 μL. Samples were incubated with primary antibodies at 4 °C overnight with gentle rotation: antibody against the NBCn1 N-terminal (kindly provided by Prof. Jeppe Praetorius, Aarhus University) 2 μL/mg protein, antibody against RACK1 (BD Biosciences, cat. #610177) 1.25 μg/mg protein, IgG isotype control (Cell Signaling, cat. #2729) 1 μg/mg protein, IgM isotype control (eBioscience, cat. #14475282) 1 μg/mg protein). Dynabeads Protein G (Invitrogen, sat. #10004D) were washed twice for 10 min at 4 °C in 1 ml lysis buffer while rotating and immune complexes were incubated with 1.5 mg beads for 45 min at 4 °C with gentle rotation. Dynabeads with bound protein were washed 5 × 5 min in 500 μL lysis buffer, boiled for 5 min at 95 °C with 80 μL X2 sample buffer, whirly mixed thoroughly, and incubated on ice for 30 min. Proteins were separated using SDS-PAGE and analysed by Western blotting as above. The data shown are representative of results from 4 such experiments while 3 additional experiments showed inconsistent results, presumably reflecting a dynamic interaction sensitive to, e.g. confluence and cell passage number.

### siRNA transfection

Two siRNA’s directed against RACK1 (NM_006098) and corresponding mock control oligo were purchased from OriGene (OriGene Technologies, Rockville, MD, #SR307048 and #SR30004). Cells were 50%-60% confluent upon transfection: medium was replaced with fresh growth medium without penicillin/streptomycin, and cells were transfected with 20 nM siRNA using Lipofectamine 2000 (Invitrogen, #11668-019) mixed in growth medium without serum and penicillin/streptomycin. 24 h after transfection, medium was replaced with full growth medium and 72 h after transfection, cells were processed for cell surface biotinylation (as described above but without polarization) followed by Western blotting.

### FLAG-constructs and FLAG co-immunoprecipitation

Four different versions of the rat NBCn1 (rNBCn1-D, NM_058211.2) 137 amino acid long C-terminus were amplified by PCR: full length C-terminus consisting of NBCn1 C-terminal amino acids 1118–1254 (137) and truncated versions of the C-terminus consisting of NBCn1 C-terminal amino acids 1118–1239 (122), 1118–1158 (41) and 1118–1140 (22). Primers are given below. PCR-products were analyzed by gel electrophoresis and single band products were excised and subjected to DNA gel purification. Products were inserted into the pFLAG-CMV-2 expression plasmid using the In-Fusion HD Cloning Kit (Takara Bio USA) to construct N-terminally FLAG-tagged products. pFLAG-CMV-2 was linearized using EcoRI and KpnI. The resulting four plasmids were verified by restriction cutting and sequencing. Plasmids were transiently transfected into HEK293 cells at 60–80% confluence using Lipofectamine 3000 (Invitrogen). Medium was replaced with fresh growth medium after 6 h incubation with transfection mixture. Twenty-four h after transfection cells were lysed and subjected to co-IP and Western blotting as described in the section “Native co-immunoprecipitation of NBCn1 and RACK1”. FLAG was precipitated using anti-FLAG M2 antibody (Sigma-Aldrich, #F1804) at 1 µg per mg protein in the IP sample. FW (all constructs) 5′-GCGGCCGCGGAATTCcccatgatggttcttgcattagtc-3′, Rev (137) 5′-TCTAGAGTCGACTGGcaatgaagtttcagcatccatg-3′, Rev (122) 5′-TCTAGAGTCGACTGGtgtgagtgtgacaataaatttcgaa-3′, Rev (41) 5′-TCTAGAGTCGACTGGcttctccttcttcttcttgtcatc-3′ and Rev (22) 5′-TCTAGAGTCGACTGGtttcttactttctggcatgagg-3′.

### NBCn1 internalization assay

MDCK-II cells cultured on Transwell filters for 4 days were labeled with 0.2 mg/ml impermeable EZ-Link Sulfo-NHS-SS-Biotin, only added to the basolateral side of the filters, for 30 min at 4 °C, followed by quenching as described under “Cell surface biotinylation assay”. Except for a total surface control and a 0 min control, which remained at 4 °C in cold CO_2_-independent medium (CIM, Gibco #18045-088), cells were transferred to 37 °C pre-heated growth medium to allow protein internalization for the indicated time points. Subsequently, cells were returned to 4 °C to halt internalization and were washed 3 × 20 min with gentle shaking in a cold stripping buffer (75 mM reduced L-glutathione, 75 mM NaCl, 75 mM NaOH, 1% BSA, 10 mM ethylenediaminetetraacetic acid (EDTA), final pH 8.6) to remove biotin from non-internalized proteins. Finally, cells were washed several times in cold PBS, and either fixed in 4% PFA for immunofluorescence analysis or lysed in RIPA buffer and processed for cell surface biotinylation followed by Western blotting as described above.

### Data and statistical analyses

Data are shown as representative individual experiments or presented as mean with SEM error bars. Significance between two groups was tested using Student *t* test and one- or two-way ANOVA, with Bonferroni or Dunnett’s post-test for comparison of more than two groups.

### Data availability

The datasets generated and analyzed during the study are available from the corresponding authors on reasonable request.

## Electronic supplementary material

Supplementary Information
