# Supplementary material for: Trafficking, localization and degradation of the Na+,HCO3− co-transporter NBCn1 in kidney and breast epithelial cells
Source: Sci Rep. 2018 May 9;8:7435. doi: 10.1038/s41598-018-25059-7 (PMC5943355; doi:10.1038/s41598-018-25059-7)
Supplement: Supplementary file 1 — Supplementary Information [file 41598_2018_25059_MOESM1_ESM.pdf]

Supplementary figures and figure legends for:

Trafficking, localization and degradation of the Na^+,HCO_3^- co-transporter NBCn1 in kidney and breast epithelial cells

Christina Wilkens Olesen, Jens Vogensen, Ida Axholm, Marc Severin, Julie Schnipper, Isabella Skandorff Pedersen, Jakob Hjorth von Stemann, Jacob Morville Schrøder, Dan Ploug Christensen[#] *and Stine Falsig Pedersen*[#]

Section for Cell Biology and Physiology, Department of Biology, University of Copenhagen, Universitetsparken 13, DK-2100 Copenhagen Ø, Denmark

[#]*Shared last authors*

Figure S1. Localization of NBCn1 in MCF-7 cells

MCF-7 cells cultured on Transwell filters for 4 days to allow polarization. Fluorescence images of NBCn1 (magenta) and NHE1 (green). Nuclei stained with DAPI (blue). Images were collected as z-stacks on a confocal microscope and shown as z-projections with corresponding xz-scans. Scale bar: 10 µm. Data are representative of 3 independent experiments.

Figure S2. Deletion of the C-terminal tail, but not loss of the PDZ-binding motif, abolishes NBCn1 membrane localization in MCF-7 cells

MCF-7 cells were transiently transfected with GFP-coupled full-length NBCn1 (GFP-NCBn1), NBCn1 with deleted C-terminal (ΔCt-NBCn1), NBCn1 with a 4-amino acid deletion in the putative PDZ domain (ΔPDZ -NBCn1) or NBCn1 with mutated PDZ domain (EGSL-NBCn1), fixed and subjected to immunofluorescence analysis. The figure shows fluorescence images of F-actin (magenta), GFP (green), and nuclei are stained with DAPI (blue). Scale bar 10 µm. Data are representative of 2-3 independent experiments per construct.

Figure S3. RACK1 colocalization and proximity with NBCn1 in MCF-7 and SKBr-3cells

A. Immunofluorescence analysis of NBCn1 (magenta) and RACK1 (green) in semi-confluent MCF-7 cells. Arrows point to regions of detectable co-localization. Scale bar: 10 µm.

B. Immunofluorescence analysis of NBCn1 (magenta) and RACK1 (green) in SKBr-3 cells cultured for 1 (top) or 5 (bottom) days. Arrows point to regions of detectable co-localization, the star points to a region of apparent non-interaction close to the membrane when tight cell-cell interactions have formed. Scale bar: 10 µm. Representative of 2-3 experiments per condition. C,D. Proximity ligation assay to assess close proximity of RACK1 and NBCn1 in MCF-7 cells. Cells were incubated with primary antibodies directed against both NBCn1 and RACK1 (top) or only NBCn1 as a control (bottom). The Proximity ligation (PLA) signal appears as magenta dots. F-actin was stained with phalloidin^{488} (green). C. Quantification of PLA dots was carried out in ImageJ. PLA signals in 10 randomly chosen cells from 10 different image areas were counted, and the average PLA signal per cell is shown in the bar graph. Data are representative of 3 independent experiments. PLA data are shown as mean with SEM error bar. **, indicate $P < 0.01$. Student's *t* test.

Figure S4. Protein turnover of NHE1 in MCF-7 cells.

MCF-7 cells were cultured for 48 h before they were treated with 100 µg/ml CHX for the indicated time. Cells were lysed and processed for Western blotting. A. representative Western blots of NHE1 degradation +/- CHX. ß-actin was used as a loading control. B. Quantification of data as in A. Values are normalized to 0 h for each treatment and are representative of 3-5 independent

experiments per time point and condition. Western blot data are shown as means with SEM error bars. *: comparison of values under the same treatment; #: comparison of values at the same condition but under different treatments. * / #, ** / ##, *** / ### and **** / #### indicate $P < 0.05$, $P < 0.01$, $P < 0.001$ and $P < 0.0001$, relative to the initial value for that group (stars) or to the corresponding non-CHX-treated controls at the same time point (hash-tags). Two-way ANOVA with Bonferroni post-test. C. MCF-7 cells were cultured for 48 h before they were treated +/- 100 µg/ml CHX for the indicated time. Cells were lysed and processed for immunoblotting Representative immunoblots showing total PARP or cleaved PARP (c-PARP) expression. ß-actin was used as a loading control. Data are representative of 3-5 independent experiments.

Fig S5. NBCn1 degradation in MDCK-II cells is slowed down by lysosomal inhibition.

MDCK-II cells were exposed to 2.5 µg/ml CHX or 2.5 µg/ml CHX + 10 nM ConA for indicated time-periods under standard cell culture conditions. Cells were lysed and lysates were subjected to SDS-PAGE and Western blotting against NCBn1, Cyclin B1 and β-actin. A. Representative Western blots. B. Densitometry of data as in A. Data are represented as means with SEM error bars. Data are based on 3 independent experiments. *, $P < 0.05$ vs. CHX exposure. Two-way ANOVA with Bonferroni's multiple comparisons test.

Figure S1

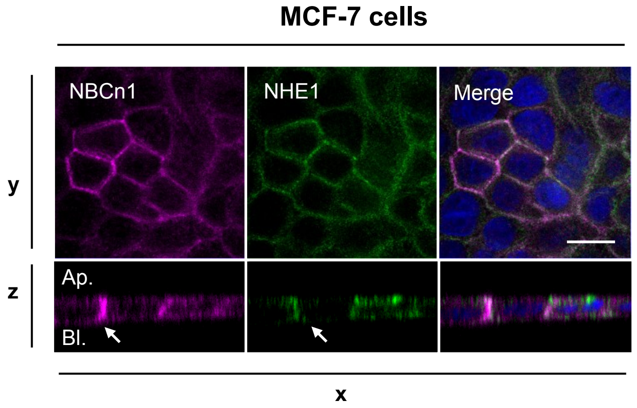

Figure S2

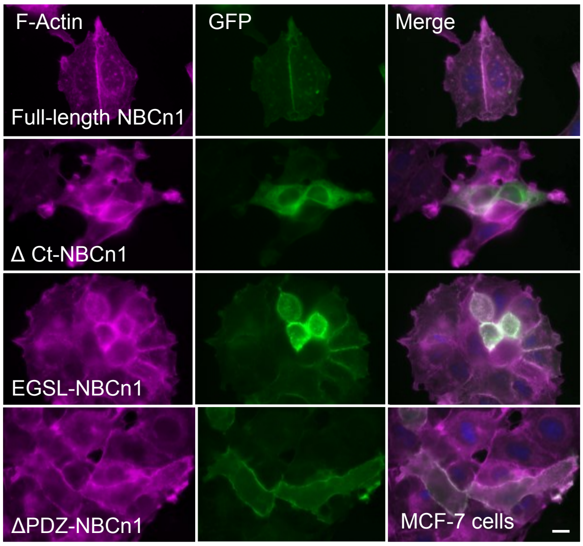
F-Actin
GFP
Merge
Full-length NBCn1
Δ Ct-NBCn1
EGSL-NBCn1
ΔPDZ-NBCn1
MCF-7 cells

Figure S3

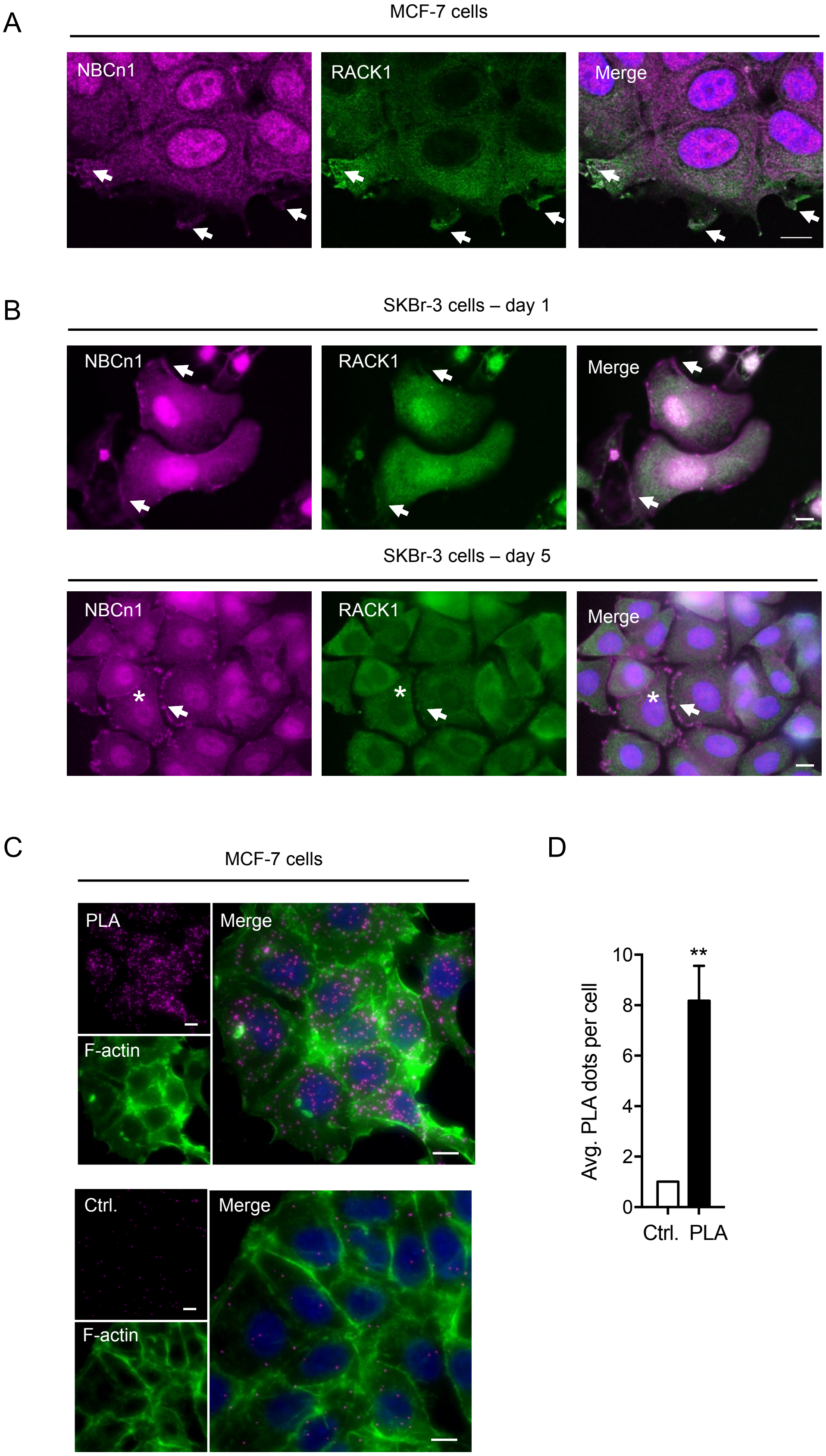
A
MCF-7 cells
NBCn1
RACK1
Merge
B
SKBr-3 cells – day 1
NBCn1
RACK1
Merge
SKBr-3 cells – day 5
NBCn1
RACK1
Merge
*
C
MCF-7 cells
PLA
Merge
F-actin
Ctrl.
Merge
F-actin
D
Avg. PLA dots per cell
0
2
4
6
8
10
**
Ctrl.
PLA

Figure S4

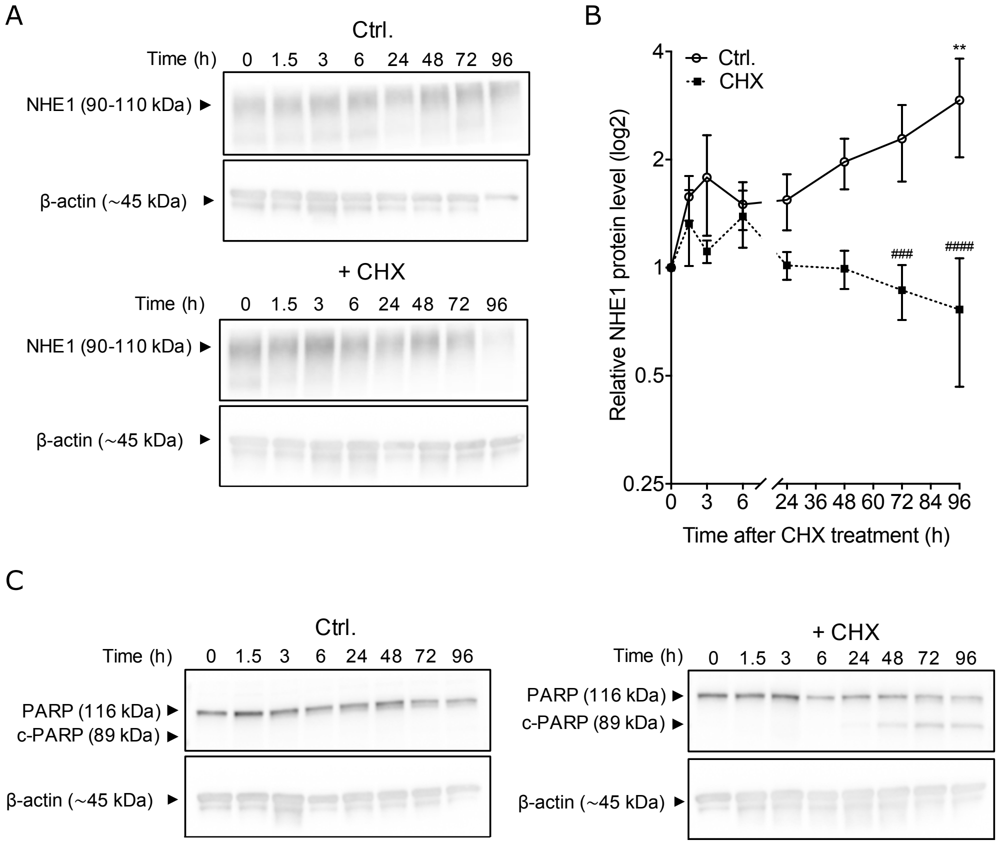

A
Ctrl.
Time (h) 0 1.5 3 6 24 48 72 96
NHE1 (90-110 kDa) ►
β-actin (~45 kDa) ►
+ CHX
Time (h) 0 1.5 3 6 24 48 72 96
NHE1 (90-110 kDa) ►
β-actin (~45 kDa) ►
B
Ctrl.
CHX
**
###
####
Relative NHE1 protein level (log2)
4
2
1
0.5
0.25
0 3 6 24 36 48 60 72 84 96
Time after CHX treatment (h)
C
Ctrl.
Time (h) 0 1.5 3 6 24 48 72 96
PARP (116 kDa) ►
c-PARP (89 kDa) ►
β-actin (~45 kDa) ►
+ CHX
Time (h) 0 1.5 3 6 24 48 72 96
PARP (116 kDa) ►
c-PARP (89 kDa) ►
β-actin (~45 kDa) ►

Figure S5

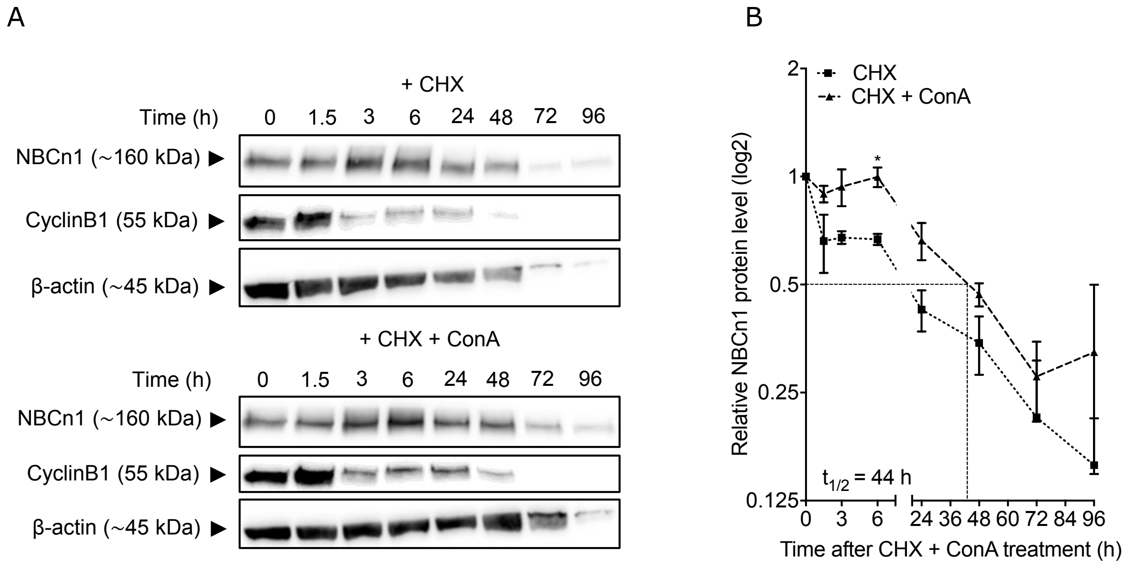
A
+ CHX
Time (h)
0
1.5
3
6
24
48
72
96
NBCn1 (~160 kDa) ▶
CyclinB1 (55 kDa) ▶
β-actin (~45 kDa) ▶
+ CHX + ConA
B
CHX
CHX + ConA
Relative NBCn1 protein level (log2)
2
1
0.5
0.25
0.125
t1/2 = 44 h
0 3 6 24 36 48 60 72 84 96
Time after CHX + ConA treatment (h)
